# The emerging landscape of dynamic DNA methylation in early childhood

**DOI:** 10.1186/s12864-016-3452-1

**Published:** 2017-01-05

**Authors:** Cheng-Jian Xu, Marc Jan Bonder, Cilla Söderhäll, Mariona Bustamante, Nour Baïz, Ulrike Gehring, Soesma A. Jankipersadsing, Pieter van der Vlies, Cleo C. van Diemen, Bianca van Rijkom, Jocelyne Just, Inger Kull, Juha Kere, Josep Maria Antó, Jean Bousquet, Alexandra Zhernakova, Cisca Wijmenga, Isabella Annesi-Maesano, Jordi Sunyer, Erik Melén, Yang Li, Dirkje S. Postma, Gerard H. Koppelman

**Affiliations:** 1 Department of Pulmonology, GRIAC Research Institute, University of Groningen, University Medical Center Groningen, Groningen, The Netherlands; 2 Department of Genetics, University of Groningen, University Medical Center Groningen, Groningen, The Netherlands; 3 Department of Biosciences and Nutrition, Karolinska Institutet, Stockholm, Sweden; 4 Department of Women’s and Children’s Health, Karolinska Institutet, Stockholm, Sweden; 5 ISGlobal, Centre for Research in Environmental Epidemiology, Barcelona, Spain; 6 Centre for Genomic Regulation, Barcelona Institute of Science and Technology, Barcelona, Spain; 7 Universitat Pompeu Fabra (UPF), Barcelona, Spain; 8 CIBER Epidemiología y Salud Pública (CIBERESP), Madrid, Spain; 9 Epidemiology of Allergic and Respiratory Diseases Department (EPAR), Sorbonne Université, UPMC Univ Paris 06, INSERM, Pierre Louis Institute of Epidemiology and Public Health, Saint-Antoine Medical School, Paris, France; 10 Institute for Risk Assessment Sciences, Utrecht University, Utrecht, The Netherlands; 11 Department of Allergology-Centre de l’Asthme et des Allergies, Hôpital d’Enfants Armand Trousseau, Assistance Publique-Hôpitaux de Paris, Paris, France; 12 Department of Clinical Science and Education, Stockholm South General Hospital, Karolinska Institutet, and Sachs’ Children’s Hospital, SE-118 83 Stockholm, Sweden; 13 Folkhälsan Institute of Genetics and Research Programs Unit, University of Helsinki, Helsinki, Finland; 14 IMIM (Hospital del Mar Medical Research Institute), Barcelona, Spain; 15 University Hospital, Montpellier, France; 16 MACVIA-France, Contre les Maladies Chroniques pour un VIeillissement Actif en France, European Innovation Partnership on Active and Healthy Ageing Reference Site, Paris, France; 17 INSERM, VIMA: Ageing and chronic diseases. Epidemiological and public health approaches, U1168 Paris, France; 18 UVSQ, UMR-S 1168, Université Versailles St-Quentin-en-Yvelines, Versailles, France; 19 Department of Paediatric Pulmonology and Paediatric Allergy, Beatrix Children’s Hospital, GRIAC Research Institute, University of Groningen, University Medical Center Groningen, Groningen, The Netherlands; 20 Department of Paediatric Pulmonology and Paediatric Allergy, University of Groningen, University Medical Center Groningen, Beatrix Children’s Hospital, GRIAC Research Institute, Groningen, The Netherlands

**Keywords:** DNA methylation, Aging, Methylation quantitative trait loci, Maternal smoking

## Abstract

**Background:**

DNA methylation has been found to associate with disease, aging and environmental exposure, but it is unknown how genome, environment and disease influence DNA methylation dynamics in childhood.

**Results:**

By analysing 538 paired DNA blood samples from children at birth and at 4–5 years old and 726 paired samples from children at 4 and 8 years old from four European birth cohorts using the Illumina Infinium Human Methylation 450 k chip, we have identified 14,150 consistent age-differential methylation sites (a-DMSs) at epigenome-wide significance of *p* < 1.14 × 10^−7^. Genes with an increase in age-differential methylation were enriched in pathways related to ‘development’, and were more often located in bivalent transcription start site (TSS) regions, which can silence or activate expression of developmental genes. Genes with a decrease in age-differential methylation were involved in cell signalling, and enriched on H3K27ac, which can predict developmental state. Maternal smoking tended to decrease methylation levels at the identified da-DMSs. We also found 101 a-DMSs (0.71%) that were regulated by genetic variants using *cis*-differential Methylation Quantitative Trait Locus (*cis*-dMeQTL) mapping. Moreover, a-DMS-associated genes during early development were significantly more likely to be linked with disease.

**Conclusion:**

Our study provides new insights into the dynamic epigenetic landscape of the first 8 years of life.

**Electronic supplementary material:**

The online version of this article (doi:10.1186/s12864-016-3452-1) contains supplementary material, which is available to authorized users.

## Background

DNA methylation is the most extensively studied epigenetic mechanism. An individual’s DNA methylation profile is not static but subject to dynamic changes induced by genetic [1, 2], environmental [3, 4] and stochastic factors during ageing. Although some age-related epigenetic changes have been identified [5–9], how genetic and environmental factors influence methylation-change with ageing is not yet well understood.

To answer these questions, we performed an epigenome-wide longitudinal DNA methylation study of 632 children across four European population-based birth cohorts participating in the MeDALL (Mechanisms of Development of ALLergy) consortium epigenetic study [10] using the Illumina Infinium HumanMethylation450 BeadChip (HM450) array. We compared 269 children at ages 0 and 4/5 years, by investigating 538 paired samples of cord blood and peripheral blood DNA, and another set of 363 children at ages 4 and 8 years (726 paired samples of peripheral blood DNA). We first identified a set of overlapping and consistent age-differential methylation sites (a-DMSs) in each group and looked at their functions. Then we linked genetic variation and maternal smoking during pregnancy with the changes in these a-DMSs over time to see how genetic and environmental factors regulate dynamic changes in methylation. Finally, we investigated if genes annotated to these a-DMSs were enriched for disease-associated genes; we linked these a-DMSs to asthma, the chronic disease with the highest prevalence in childhood.

## Results

### Identification of a-DMSs

**Fig. 1:**
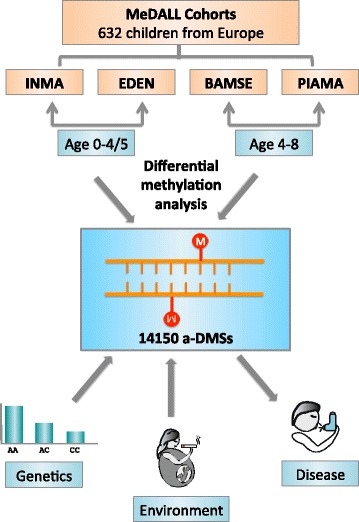
Study design and structure of this paper. We collected cord blood and peripheral blood samples from 632 children from four European birth cohorts: INMA, EDEN, BAMSE and PIAMA. Differential methylation analysis on different age groups resulted in 14,150 consistent age-differential methylation sites (a-DMSs). We then linked the methylation change of these a-DMSs to genetics by *cis*-dMeQTL, to environmental exposure from maternal smoking, and to diseases, specifically asthma

We first applied differential methylation analysis on 439,306 cytosine-phosphate-guanine (CpG) sites that passed our quality control (see Methods) in 538 paired samples from the INMA (Spain) and EDEN (France) birth cohorts (ages 0 and 4/5) and in 726 paired samples from the BAMSE (Sweden) and PIAMA (Netherlands) birth cohorts (ages 4 and 8), (Fig. 1, Additional file 1: Table S1). The overall correlations of methylation levels between two time points in the four birth cohorts are shown in Additional file 2: Figure S1. In the pooled analysis of all cohorts adjusting for cell-type-composition [11], 15,529 significant a-DMSs were identified in both age groups surpassing the 5% Bonferroni corrected threshold (*P* < 1.14 × 10^−7^) (Additional file 1: Figures S2 and S3). Of these 15,529 a-DMSs, 9,704 (62.5%) showed a consistent decrease in methylation with age (decrease in age-differential methylation sites, da-DMSs), whereas 4,446 (28.6%) showed a consistent increase in methylation with age (increase in age-differential methylation sites, ia-DMSs). Only 1,379(8.9%) of the CpG sites showed opposite directions in their methylation changes between the younger and older comparison sets (Additional file 1: Table S2). The complete list of 14,150 consistent da-DMSs and ia-DMSs, and their annotation, is presented in Additional file 3. Comparing our a-DMS results with those from two independent, paediatric, age-related methylation studies [7, 8], we see more than 50% overlap in the CpG sites identified (Additional file 1: Table S3). We observed that some of our consistent da-DMSs were located in clusters, probably reflecting age associated differential methylated regions rather than one site.

The most significant ia-DMSs, cg01511232 (p_0-4/5_ = 7.92 × 10^−148^, p_4-8_ < 2.25x10^−308^) and cg22398226 (p_0-4/5_ = 1.66 × 10^−149^, p_4-8_ = 5.09x10^−307^) are located on the chromosome 4q32.1, near the gene *LRAT*. Mutations in this gene have been associated with early onset retinal dystrophy [12]. The top associated da-DMSs, cg16069986 (p_0-4/5_ = 6.28 × 10^−114^, p_4-8_ < 2.25x10^−308^) and cg16312514(p_0-4/5_ = 1.67 × 10^−106^, p_4-8_ < 2.25x10^−308^) on chromosome 11q13.4 region were annotated in *SHANK2*. Mutations in this *SHANK2* synaptic scaffolding gene have been associated with neurodevelopmental disorders and autism [13].

### Characterization of da-DMSs and ia-DMSs

**Fig. 2:**
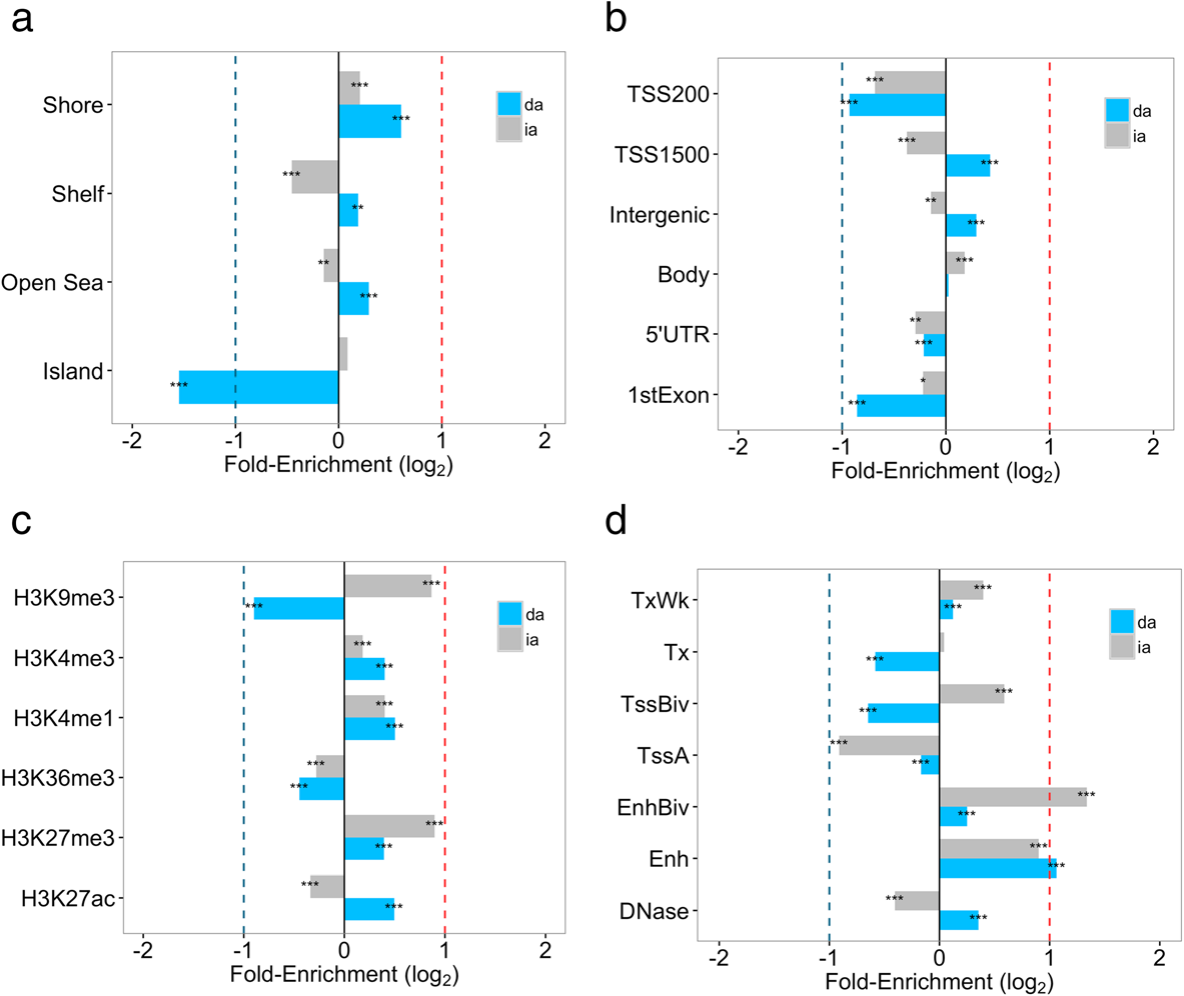
Molecular enrichment of a-DMSs for regulatory features. Fold enrichment of 9,704 da-DMSs (*blue bar*) and 4,446 ia-DMSs (*grey bar*). **a** CpG islands, shore, shelf and open sea. **b** Gene regions including: within 1.5 kb upstream of the transcription start site (TSS1500), the 5' untranslated region (5′UTR), the first gene exon, the gene body, the 3' UTR or intergenic region. **c** Predicted gene expression regulatory regions based on histone modifications derived from Roadmap epigenomics blood data. **d** Predicted chromatin state based on Roadmap epigenomics blood data. DNase: DNase I hypersensitive site, Enh: Enhancers, EnhBiv: Bivalent enhancer, TssA: Active TSS, TSSBiv: Bivalent/poised TSS, Tx: Strong transcription, TxWk: Weak transcription. The presented fold enrichments are from 1,264 samples by *χ* ^2^ test and are relative to all 437,792 CpG sites tested (y-axis); * 1 × 10^−3^ ≤ *P* < 0.01, ** 1 × 10^−6^ ≤ *P* < 1 × 10^-3,^ *** *P* < 1 × 10^−6^

We next examined the enrichment of the 9,704 da-DMSs and 4,446 ia-DMSs in genomic regions and their predicted roles in regulating gene expression using histone modifications and chromatin state data from the Roadmap Epigenomics Project [14]. ia-DMSs were situated in distinctly different functional domains compared to da-DMSs, which is consistent with previous findings [9, 15]. Thus, compared to all the CpG sites we tested, ia-DMSs were enriched in CpG shores (Fig. 2a) and gene bodies (Fig. 2b), while da-DMSs were enriched in open seas, shelves, shores, intergenic regions and transcription start site (TSS) 1500 regions. ia-DMSs were also enriched in transcriptional repression histone modification marker (H3K9Me3), while da-DMSs were depleted for this marker (Fig. 2c). Both da-DMSs and ia-DMSs were enriched in enhancers and bivalent enhancers (Fig. 2d). ia-DMSs were also enriched in bivalent TSS regions (Fig. 2d), which are thought to silence or activate expression of developmental genes [16]. In contrast, da-DMSs were enriched in H3K27ac, which separates active from poised enhancers and predict developmental state [17].

We next performed pathway analysis by GeneNetwork [18] on genes located in close proximity to DMSs probes. In total, 3559 genes were annotated to be located near ia-DMSs probes and 7130 genes near the da-DMSs probes. 1940 of these genes were overlapping across the two groups, suggesting complex regulation of these genes during development. The pathway analysis of da-DMSs specific genes using gene co-expression indicated enrichment of Reactome pathways involved in cell signalling, including signalling by nerve growth factors, platelet derived growth factors, neurotrophin signalling and many others (Additional file 4). The corresponding Mouse Genome Informatics (MGI) phenotypes pointed to cell migration, growth retardation and various abnormal tissue morphologies. The ia-DMS specific genes were enriched for developmental genes, axon guidance, regulation of insulin secretion and energy metabolism. The enriched MGI phenotypes were related to neonatal and postnatal lethality. This indicates that in early age methylation regulates the activity of genes, involved in basic processes of development and metabolism.

Next, we inspected methylation dynamics of some well-known developmental genes and found a lot of CpGs sites which were annotated to them, such as growth differentiation factors(*GDFs*), Bone morphogeneic proteins(*BMPs*), Wnt signalling pathway (*Wnt*) gene, Paired box genes(*Pax*) and homeotic genes (*Hox*) gene etc. Among them, the 5′ *HOXD* cluster [19] that contains genes important for limb development. The 5′ *HOXD* cluster consists of five genes (*HOXD9* to *HOXD*13). We found eight ia-DMSs with low methylation status within the cluster (Additional file 1: Table S4), seven of which were localized in CpG islands encompassing the five promoters and three first exons. In contrast, Meis homeobox 1 (cg12055515, *MEIS1*, *P*_0-4/5_ = 7.95 × 10^−17^, *P*_4-8_ = 1.77 × 10^−11^), a limb development regulator that forms complexes with both *HOXD*9 and *HOXD*10 [19], displays a different methylation pattern. Cg12055515 lies in the gene body of *MEIS1* and its methylation level decreases during ageing (Additional file 1: Table S5).

### Genetic variation associates with longitudinal DNA methylation changes

The availability of paired samples from four different cohorts gave us the possibility to investigate the effect of genetics on methylation dynamics in a longitudinal way. We examined whether genetic variations were associated with longitudinal DNA methylation changes in childhood using *cis*-differential Methylation Quantitative Trait Locus (*cis*-dMeQTL) mapping. We associated SNPs within ±250 kb of our 14,150 a-DMSs in GWAS data available for 230 children from BAMSE and PIAMA in the 4–8 years-old comparison set for discovery and from 114 children in the INMA 0–4/5 years-old comparison set for replication (Additional file 1: Table S1). We discovered 7,316 SNP-CpG pairs on 866 aDMSs with significant *cis*-dMeQTLs in the older comparison set (FDR < 0.05), and 3,534 SNP-CpG pairs (48.3%) were replicated in the younger comparison set (same direction of effects and nominal *P* < 0.05). 113 potentially false-positive dMeQTLs were removed due to SNP-under-the-probe effects [20]. The remaining 3,421 (46.8%) SNP-CpG pairs are listed in Additional file 5, consisting of 2734 SNPs regulating 101 a-DMSs (60 da-DMSs and 41 ia-DMSs) that changed with age and showed methylation change was partly under genetic control. To understand these dMeQTLs, we tested if these 3,421 SNP-CpG pairs were MeQTLs at age 4 and 8 years cross-sectionally. Most SNP-CpG pairs showing dMeQTL were significant MeQTLs when analysed cross-sectionally at both ages (Additional file 3). The most significant MeQTL rs7522439-cg00025357, which are on chromosome 1p36.13, close to gene *PADI3* . By checking the BIOS QTL browser (http://genenetwork.nl/biosqtlbrowser/), we also found the significant *cis*-MeQTL (*P* = 3.3 × 10^−310^) in methylation data from adult blood DNA samples, which is line with the recent findings that genetic effects on methylation levels are stable [21]. However, we also found three CpG sites cg00804078, cg02872436 and cg14956327 which were annotated to the *DDO* (D-aspartate oxidase) gene showing significant *cis*-MeQTL, but did not show significant *cis*-MeQTL in the BIOS QTL database. This may indicate an age specific MeQTL effect since D-aspartate content in brain is decreased during adulthood [22].

**Fig. 3:**
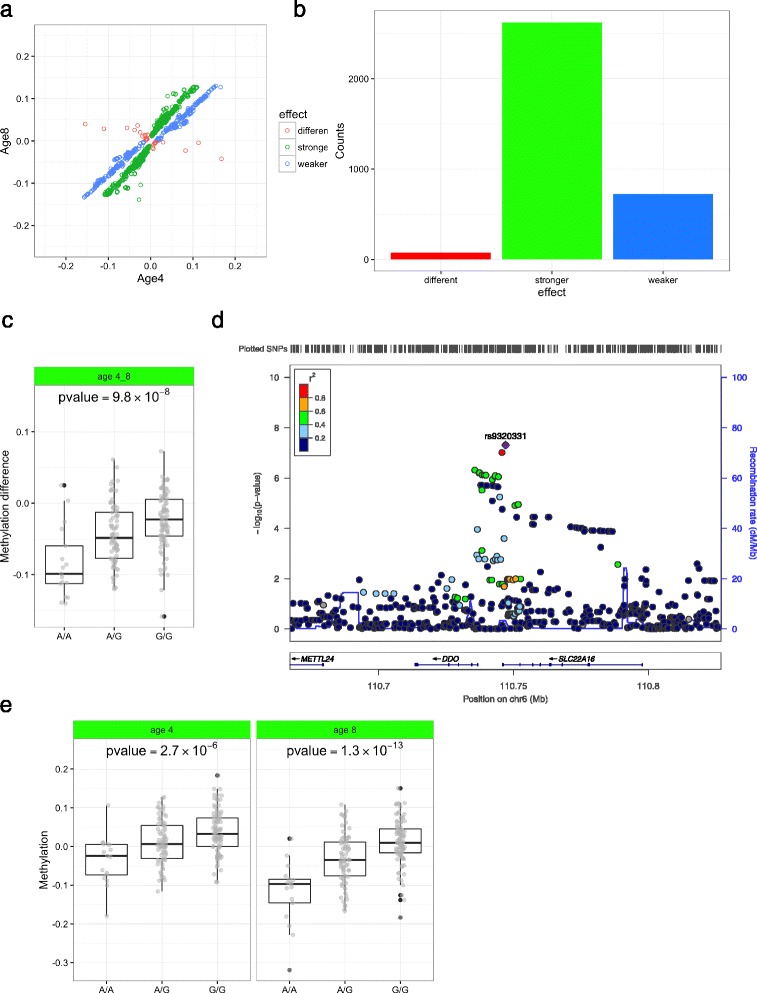
Genetic variation associated with longitudinal DNA methylation changes by *cis*-dMeQTL study. **a** The effect sizes of MeQTL at age 4 years against those at age 8 years of 3421 SNP-CpG pairs in the age 4-8 group. Almost all the dots lie in the first and third quadrants, suggesting that the effect size of genetic factors on methylation from most of the SNP-CpG pairs followed the same direction at both ages. Blue dots represent SNP-CpG pairs with a significantly weaker effect size at age 4 than at age 8, whereas green dots represent a stronger effect, and red dots represent a different direction of effect. **b** Bar plot of 3421 SNP-CpG pairs. There are 725 SNP-CpG pairs showing a weaker effect at age 4 than age 8 (*blue bar*), 3,348 SNP-CpG pairs showing stronger effect (*green bar*) and 73 SNP-CpG pairs showing a different effect (*red bar*). **c** Boxplot of dMeQTL of rs9320331-cg00074818. 181 PIAMA samples have been used to illustrate this dMeQTL. **d** Regional dMeQTL association of cg00074818 results for the age 4–8 group. Plots were generated using LocusZoom [48]. The LD estimates are color-coded as a heatmap from dark blue (0 ≥ *r* ^2^ > 0.2) to red (0.8 ≥ *r* ^2^ > 1.0) Regional plot shows-log_10_ *P* of all SNPs surrounding SNP rs9320331, and the degree of linkage disequilibrium between all SNPs and lead SNP rs9320331. SNPs with lower *P*-values span *DDO* and *SLC22A18* genes within a recombination boundary. **e** Boxplot of MeQTL of rs9320331-cg00074818 at age 4 and age 8 cross-sectionally

Furthermore, we compared the MeQTL-effect size at ages 4 and 8 (Fig. 3a), and found that almost all effects of genetic factors on methylation had the same direction at both ages (3,348 SNP-CpG pairs; 97.9%), i.e., genetic effects on methylation remained stable with ageing. Among these 3,348 SNP-CpG pairs, 725 had a significantly larger effect and 2,623 had a significantly smaller effect at age 4 years than at age 8 (Fig. 3b, 1-sample proportion test, *P* = 4.8 × 10^−236^). An example of this pattern (Figs. 3c and d) is shown for the rs9320331_cg00804078 SNP-CpG pair in the *DDO* (D-aspartate oxidase) gene, which showed a MeQTL at both ages 4 and 8 (Fig. 3e), with a stronger effect at age 8(beta = 0.058, *P* = 1.3 × 10^−13^) compared to the effect at age 4 (beta = 0.032, *P* = 2.7 × 10^−6^).

### Maternal smoking during pregnancy associates with longitudinal DNA methylation changes

**Fig. 4:**
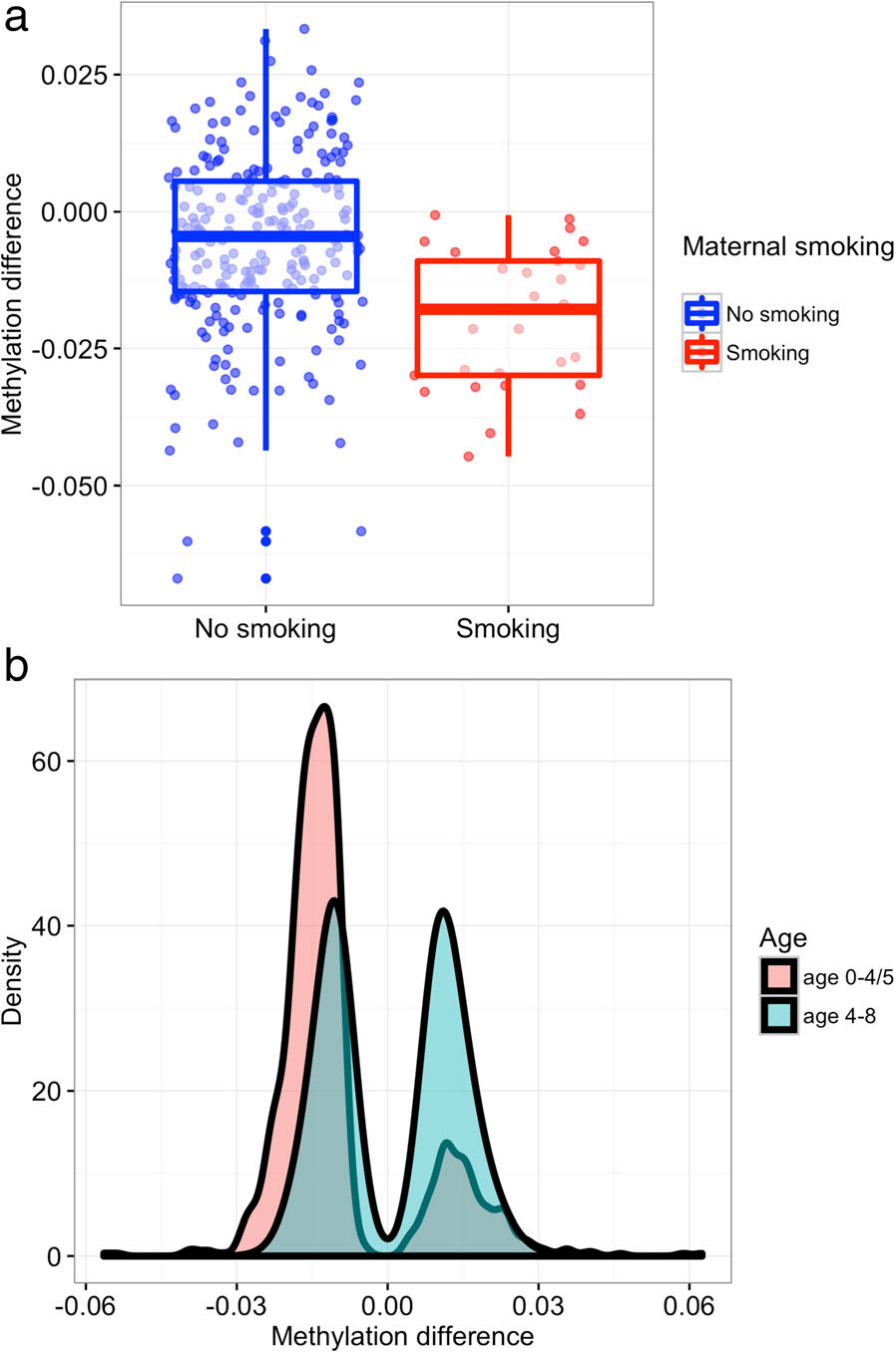
The effect of maternal smoking on methylation change. **a** The effect of maternal smoking on methylation change of cg09836827 (*VWF*) in the age 0–4/5 group. Maternal smoking shows an enhanced effect on decreasing methylation levels since the decrease from age 0 to age 4/5 years old of cg09836827 in the exposed group is significantly larger compared to the non-exposed group. **b** Density plot of effect size of all nominally significant a-DMSs (*P* < 0.05) with maternal smoking. In the age 0–4/5 group, methylation changes tend to be negatively associated with maternal smoking since there are more negatively associated a-DMSs. In the age 4–8 group, there are similar numbers of negatively- and positively associated sites

Methylation has been found strongly associated with maternal smoking during pregnancy [3]. Here we further investigated whether methylation changes of the 14,150 a-DMSs between the 0–4/5 and 4–8 years-old comparison sets were associated with maternal smoking during pregnancy. In total, we found eight significant a-DMSs in the younger set and one in the older set (FDR < 0.05, Additional file 1: Table S6) associated with maternal smoking. These nine CpG sites mapped to five genes, *ITGA11, OR5B3, VWF, RCBTB1 and CREB5* and one pseudogene *HNRNPA3P1*. The most significant association was for the methylation change of cg09836827 at the *VWF* gene (Von Willebrand Factor), a protein-coding gene crucial for haemostasis. Both *VWF* and *ITGA11* (integrin alpha 11) are involved in regulating cell-substrate adhesion. Interestingly, all nine significant CpG sites belonged to the da-DMS group, and the association of maternal smoking with methylation changes was always negative (Additional file 1: Table S6). This means that the methylation decrease was consistently larger in the children exposed to smoke in utero compared to the non-exposed. Fig. 4a and Additional file 2: Figure S4 show that maternal smoking during pregnancy was associated with a stronger, decreasing effect on methylation change of cg09836827 (*VWF*) in those children. To underpin this, we further checked the directions of effect of all nominally significant a-DMSs (*P* < 0.05) and found that methylation changes tended to be negatively associated with maternal smoking between 0 and 4/5 years (Fig. 4b) and, moreover, that 721/971 (74.2%) a-DMSs showed a stronger decrease in methylation levels from 0 to 4/5 years (Additional file 1: Table S7). In summary, we found that maternal smoking exposure tended to more strongly decrease methylation levels at the identified da-DMSs.

### DNA methylation changes related to disease development

Early children development is considered to be the most important phase in life and also of great vulnerability to negative influences. We found that many top associated a-DMSs, and their annotated gene were linked to diseases, such as *SHANK2 (*cg16069986 and cg16312514) which is related to autism, and *CSRP3* (cg05895618) *which* is linked to cardiomyopathy*.* We hypothesised that these a-DMSs annotated genes are crucial for the growth, and dysregulation of gene expression and eventually lead to disease development. Therefore, we further investigated if genes containing a-DMSs were more likely to be associated with disease by studying the enrichment of genes on our list (Additional file 1) in the catalogue of disease-associated genes provided by the Clinical Genomic Database [23]. Indeed, we found that genes containing a-DMSs were more likely to be disease-related (16.9% compared with 14.0%, *P* = 4.9 × 10^−6^, Fisher’s exact test) (Additional file 1: Table S8). This might indicate a link between age-related methylation changes and disease.

We then investigated the associations of the a-DMSs with asthma, the most common chronic pulmonary disease in childhood, and the focus of our MeDALL study [10]. Since early-life exposures strongly influence asthma development, and may affect DNA methylation [24], we hypothesized that dynamic methylation changes occurring specifically in early childhood might be associated with asthma. We tested the association of our 14,150 a-DMSs with asthma at age 4/5 and 8 years using the MeDALL asthma definition [25]. Three CpG sites were significantly associated (FDR < 0.05) with asthma at age 4: cg22971191 in *SLC10A2* (solute carrier family 10 (sodium/bile acid cotransporter) member 2), cg18515031 in *C10orf104* (chromosome 10 open reading frame 104) and cg05712073 in *ZNF384* (Zinc Finger Protein 384). We also found one significant (FDR < 0.05) CpG site, cg02977254 in *C11orf63* (Chromosome 11 Open Reading Frame 63), to be associated with asthma at age 8 (Additional file 1: Table S9).

## Discussion

This study offers insight into the dynamic epigenetic landscape of the first 8 years of life. We report a large number of DNA methylation changes that are consistent between both our age range comparisons (0-4/5 years and 4–8 years). These changes with increasing age (da-DMSs and ia-DMSs) have distinct, only partly overlapping, genetic localizations and functional annotations. The enrichment of a-DMSs in gene bodies and in enhancer regions suggest that longitudinal changes in DNA methylation are not only acting as transcription repressors, but may also act as buffers to stabilize decisions by transcription factors, ensuring precise and robust transcription [26]. Importantly, we also demonstrate that ia-DMSs occur preferentially at developmental genes (e.g., 5′ *HOXD*) cluster and are located at bivalent chromatin domains. These chromatin domains appear to be able to repress or activate epigenetic modifications, and are thought to be important during imprinting and development. We focused on consistent methylation changes that are present between ages 0 to 4 as well as 4 to 8 years, and suggest that further analyses using even shorter time periods (i.e., infancy) may reveal further insight into the epigenetic association with specific developmental periods of a child. For the first time, we have linked genome-wide genetic variants with longitudinal methylation changes by performing *cis*-dMeQTL analysis. We find that 0.71% (101/14150) of the differentially methylated CpG sites are regulated by genetic variation, which is compatible with genetic control over epigenetic plasticity. In most cases, MeQTL were more pronounced at age 8 than at age 4, indicating stronger genetic regulation later in childhood, and possibly linking genetic regulation of methylation with ageing. Zooming in on one dMeQTL, rs9320331-cg00804078 located on chromosome 6q21 close to *DDO* gene (Fig. 3), we observed relationships between this SNP, gene expression and methylation by combining our data with public data (Additional file 2: Figure S5). Methylation of cg00804078 was reported to be negatively correlated with *DDO* expression in 1,264 monocyte samples [9], while SNP rs9320331 has been detected as an eQTL of *DDO* mRNA in whole blood^27^ and other tissues [27]. The expression of the *DDO* enzyme has been reported to control the rate of brain-ageing processes by decreasing D-aspartate levels [28]. Therefore, a plausible mechanism for the observed MeQTL is that SNPs regulate the expression of *DDO* through DNA methylation to ultimately control the rate the brain ages. In our data, we observed a significantly lower methylation of cg00804078 from age 4 to 8, contributing to higher *DDO* expression and lower D-aspartate levels, and corroborating earlier findings of lower D-aspartate levels with ageing [29]. Moreover, we did not observe significant *cis*-MeQTL of cg00804078 in adult data by checking BIOS QTL browser. It may indicate that epigenetic mediation of genetic risk [30] for *DDO* expression is age specific and only plays a role in childhood.

To assess the role of an important environmental factor, we examined longitudinal changes in DNA methylation associated to maternal smoking during pregnancy and found association with decreasing methylation levels from birth up to age 4/5. Since it has been suggested that decreasing methylation is associated with ageing [6], our findings may indicate that maternal smoking during pregnancy has an immediate effect on ageing by decreasing methylation levels. However, we did not find this “decreasing methylation” effect in our 4–8 year-old set. This shift may indicate that the in utero effect of maternal smoking is most crucial during the first years of life. We further tested the effect of smoking on Horvath’s “epigenetic clock” [5], which predicts age based on DNA methylation data and statistical learning, but found no significant effect (Additional file 2: Figure S6). This may be because the “epigenetic clock” was trained on adult samples, making it less accurate for age predictions based on DNA methylation in children (Additional file 2: Figure S7).

The DOHAD (Developmental Origins of Health and Disease) hypothesis proposes that early human development affects the risk of chronic, non-communicable diseases in later life [31]. Early life development may be expressed in epigenetic mechanisms, such as DNA methylation, and this may have long-lasting effects over an individual’s life. We reasoned that the set of a-DMSs that change in the first 8 years of life could be used to test for enrichment with disease genes. One interesting observation that came out of this analysis is that childhood a-DMS-genes were more likely to be disease-linked. We therefore tested the association of identified 14150 a-DMSs with asthma, which often starts in early life and is the most prevalent chronic disease in childhood. We identified four a-DMSs associated with asthma at ages 4 and 8, a finding that warrants replication. We propose that future studies could assess the association of a-DMSs with other chronic, non-communicable diseases originating in early childhood.

A major strength of our study is the combination of four different European birth cohorts of general population samples. Although children from more highly educated parents may be overrepresented in our study [32], our study represents a reasonable representation of European populations, forming a large dataset with good statistical power. Furthermore, the paired sample approach enables conclusions on longitudinal changes, and we specifically focused on childhood ageing to complement the focus on adult ageing populations in the literature.

However, our study had some limitations: the HM450 platform only covers 1.6% of all methylation sites [33], and the design of HM450 may be biased towards CpG-richer regions of the genome. DNA was derived from different blood cell types: cord blood at age 0 versus peripheral blood at older ages; this could be a potential confounder. We therefore applied a cell-type correction [11] and only used findings consistent over the two comparison sets to support our conclusions.

## Conclusions

In this research, we observed dynamic DNA methylation changes in the first 8 years of life, with increasing or decreasing a-DMSs having specific functional annotations and genomic localizations. We describe that a subset of these a-DMSs are under genetic control, and report how maternal smoking affects DNA methylation changes in early life. Finally, we provide evidence that the set of genes annotated to a-DMSs is enriched for disease development, and specifically show 4 CpG sites to be associated with asthma in the first 8 years of life.

## Methods

### Study population

All DNA samples were obtained from the MeDALL epigenetics study, which covers four cohorts and was designed to investigate the development of asthma and allergy using a paired case-cohort design. We firstly randomly select paired samples at ages 0 and 4/5 years and age 4 and 8 years, and then added more asthma cases. This design resulted in an enrichment of subjects with asthma. The prevalence of asthma at age 4 and age 8 in all MeDALL samples are 25 and 20% respectively. Since the aim of our study was to investigate methylation in childhood ageing independent of disease status, we only selected the random samples. In total, we had 1,622 paired samples from the four European birth cohorts: BAMSE [34], EDEN [35], INMA [36] and PIAMA [32]. After random sample selection, 1,264 paired samples were used in the analysis. The prevalence of asthma at age 4 and age 8 in all random samples are 13 and 11%, respectively. The basic characteristics of the participants are given in Additional file 1: Table S1.

### DNA extraction, bisulphite treatment and DNA methylation measurement

In the MeDALL study, cord blood samples and peripheral blood samples were collected from all consenting cohort participants and DNA was extracted using the QIAamp blood kit (Qiagen or equivalent protocols), followed by precipitation-based concentration using GlycoBlue (Ambion). DNA concentration was determined by Nanodrop measurement and Picogreen quantification. 500 ng of DNA was bisulphite-converted using the EZ 96-DNA methylation kit (Zymo Research), following the manufacturer’s standard protocol. After verification of the bisulphite conversion step using Sanger Sequencing, genome-wide DNA methylation was measured using the Illumina Infinium HumanMethylation450 BeadChip. After normalization of the concentration, the samples were randomized to avoid batch effects, and all paired samples were hybridized on the same chip. Standard male and female DNA samples were included in this step as control samples.

### Quality control and pre-processing of microarray data

DNA methylation data were pre-processed in R with the Bioconductor package Minfi [37], using the original IDAT files extracted from the HiScanSQ scanner. We had a total of 1,748 blood samples from four birth cohorts in the MeDALL epigenetics study. Samples that did not provide significant methylation signals in more than 10% of probes (detection *P* = 0.01) were excluded from further analysis. Samples were also excluded in cases of low staining efficiency, low single base extension efficiency, low stripping efficiency of DNA from probes after single base extension, poor hybridization performance, poor bisulphite conversion and high negative control probe staining. Further, we used the 65 SNP probes to check for concordances between paired DNA samples from the sample individual and assessed the methylation distribution of the X-chromosome to verify gender. Paired samples with Pearson correlation coefficients <0.9 were regarded as sample mix-ups and were excluded from the study. In total, we excluded 16 samples due to poor quality and 24 samples due to apparent sample mix-up. In probe filtering [38], we excluded probes on sex chromosomes, probes that mapped on multi-loci, the 65 random SNPs assay and probes that contained SNPs at the target CpG sites with a minor allele frequency >10%. The allele frequencies of a list of SNPs were obtained from 1000 Genomes, release 20110521 for CEU population. Finally, we implemented “DASEN” [39] to perform signal correction and normalization. After quality control, 1,708 samples and 439,306 autosomal probes remained. From these, we selected 1,264 samples in pairs from the population of randomly selected children for further analysis.

### Differential methylation analysis

Methylation levels (beta values, β) at a given CpG site were derived from the ratio of the methylated probe intensity to overall intensity (sum of methylated and unmethylated probe intensities): β is equal to M/(U + M+ α), where M is the intensity of the methylated probe, U is the intensity of the unmethylated probe, and α is the constant offset with the default value of 100. To remove bias in methylation profiles due to technical variation, we implemented a correction procedure based on 613 negative control probes [40] present in HM450K arrays because these negative control probes did not relate to biological variation. First, we implemented principal component analysis (PCA) on control probe data according to the method proposed by Zhang et al. [41]. Then, we permuted the control probe data 10000 times and applied PCA to each of these permuted datasets. We then selected principal components with a *P*-value defined to get the *P*_*(*number of var(random pc)>var(pc))/(number of permutations)_ < 10^−4^. The methylation data for each CpG were the residuals from a linear model incorporating the five significant principal components that reflected technical variation. We adjusted the residuals by cohort, gender, bisulphite conversion kit batch number, position on the array and the percentage of monocytes, B cells, NK cells, CD4+ T cells, CD8+ T cells and granulocytes predicted by Houseman *et al’s* algorithm [11]. In the age-differential-methylation analysis, the significant methylation differences of CpG Sites between two age groups (ages 0–4/5 years and ages 4–8 years) were identified by fitting a robust linear regression model. For the maternal smoking analysis, smoking during pregnancy was defined as: the mother was smoking in the last trimester of her pregnancy. The detailed definition of maternal smoking for each cohort can be found in reference [3]. Additional variables included in the final robust linear model for maternal smoking analysis were maternal age, parity and maternal education.

### Genotyping, MeQTL and dMeQTL

Genotype data from the individual cohorts were imputed to reference data of the 1000 Genomes’ CEU panel (release March 2012). Detailed information on the genotyping of BAMSE, INMA and PIAMA has already been published [42]. Subsequent quality control removed SNPs with minor allele frequency (MAF) <0.01, those with Hardy Weinberg equilibrium *P* < 1x10^−6^, genotype call rate <0.95, the minimum MACH R^2^ measure to include SNPs (rsq < 0.3) for BAMSE data and Info score < 0.3 for PIAMA and INMA data. All the genotypes were aligned to the GIANT release of 1000G to facilitate further data integration and meta-analysis by genotype Harmonizer 1.4.9 [43]. We had genotype data for 114 INMA samples, age 0-4/5 years, and for 181 PIAMA and 49 BAMSE samples, age 4–8 years.

The dMeQTL and MeQTL analysis was performed using the R package MatrixEQTL [44] using an additive linear model. To remove the effect of extreme outliers, we trimmed the methylation set using: (25th percentile -3*IQR) and (75th percentile + 3*IQR), where IQR = interquartile range. SNPs were included in the *cis*-analysis if they were located within 250 kb of the methylation probe under consideration. The methylation differences between two time points were used as molecular phenotype for dMeQTL study. The fixed-effect-model based on the inverse standard error was utilized for meta-analysing BAMSE and PIAMA dMeQTL and MeQTL results at age 4–8 by using METAL [45]. The BAMSE and PIAMA samples from the 4–8 years-old comparison set were used as discovery cohorts, and the INMA samples from the younger comparison set were used as replication. The SNP-CpG pairs were considered as significant dMeQTL in the 4–8 years-old comparison set if the *P*-value after FDR correction was <0.05 and if the *P*-value was <0.05 in the replication study in the 0–4 years-old group. We filtered the *cis*-meQTLs effects by removing SNP-CpG pairs for which the same SNP was also located in the probe, or for which the SNP was outside the probe but in linkage disequilibrium (LD) (r^2^ > 0.2) with a SNP inside the probe. We tested for LD between SNPs pairs by using 1000 Genomes’ CEU data [46].

### Functional annotation analysis

For molecular enrichment analysis, CpG sets were annotated by the Illumina HM450 manifest file (version 1.2). Annotations used were classified as gene related (TSS1500 and TSS200, regions from-1500 to–200 and-200 to the transcriptional start site, respectively; 5′ UTRs; first exons; gene bodies; 3′ UTR and intergenic (no gene annotation)) or CpG island-related (islands shores (0 to 2 kb flanking islands), shelves (2 to 4 kb flanking islands) and open sea (>4 kb from islands)). All 3,091 CpH sites were excluded in the enrichment analysis, leading to 437,792 CpG sites for the enrichment test. In addition, the epigenome Roadmap annotation was created by overlapping the histone marks and chromatin states for the 27 blood cell-types annotated in the epigenome Roadmap project with the CpG sites interrogated by the HM450 array. The raw annotation was retrieved from the Epigenome Roadmap web portal (http://egg2.wustl.edu/roadmap/web_portal/index.html). The mark states were combined over the 27 blood-related types by taking a state as present if it was in at least 1 of the 27 blood-related measurements.

In pathway and gene-set analysis of a-DMSs, we used the loci and gene definitions predicted in GREAT (Genomic Regions of Annotations Tool) [47] that assigns biological meaning to *cis* regulatory regions (CpG sites) using the single nearest gene association rule within a 100 kb window. We used GeneNetwork (http://129.125.135.180:8080/GeneNetwork/pathway.html) [18] for pathway analysis of a-DMSs.

### Methylation age and disease analysis

For the Horvath Age predictor, methylation age was determined in all cohorts using the online calculator (https://labs.genetics.ucla.edu/horvath/dnamage/). For the disease analysis, genes were linked to a disease based on the Clinical Genomic Database [23] (http://research.nhgri.nih.gov/CGD/download/, last accessed on Feb 27 2016). Age-related gene sets were annotated by the Illumina HM450 manifest file (version 1.2). For asthma analysis, we used the full MeDALL data at age 4/5 years and 8 years (asthma cases and population-based controls) to investigate the associations between CpG methylation and asthma status. The basic characteristics of participants included in the asthma study are listed in Additional file 1: Table S10. The final robust regression model was as follows: methylation levels (corrected by 5PC of control probes) ~ asthma + co-variables + cell counts. The co-variables included were cohorts, gender, bisulphite conversion kit batch number, duration of the 450 K assay, position on the array and cell count percentages of monocytes, B cells, NK cells, CD4+ T cells, CD8+ T cells and granulocytes predicted by Houseman *et al*’s algorithm [11].

## Additional files

Additional file 1: Tables S1-S10. (DOCX 1279 kb)
Additional file 2: Figures S1-S7. (DOCX 45 kb)
Additional file 3: Age-differential methylation sites in early childhood (XLSX 2094 kb)
Additional file 4: Pathway analysis of age-differential methylation sites by gene network. (XLSX 3782 kb)
Additional file 5: List of age differential Methylation Quantitative Trait Locus in early childhood (XLSX 336 kb)

## Abbreviations

a-DMS: Age-Differential Methylation Site
CpG: Cytosine-phosphate-Guanine
da-DMSs: Decrease in age-Differential Methylation Sites
dMeQTL: Differential Methylation Quantitative Trait Locus
HM450: Illumina Infinium HumanMethylation450 BeadChip
ia-DMSs: Increase in age-Differential Methylation Sites
MeDALL: Mechanisms of Development of ALLergy
MGI: Mouse Genome Informatics
TSS: Transcription Start Site

## References

[1] Lemire M, Zaidi SHE, Ban M, Ge B, Aïssi D, Germain M (2015). Long-range epigenetic regulation is conferred by genetic variation located at thousands of independent loci. Nat Commun.

[2] Gutierrez-Arcelus M, Lappalainen T, Montgomery SB, Buil A, Ongen H, Yurovsky A (2013). Passive and active DNA methylation and the interplay with genetic variation in gene regulation. Ponting CP, editor eLife.

[3] Joubert BR, Felix JF, Yousefi P, Bakulski KM, Just AC, Breton C (2016). DNA Methylation in Newborns and Maternal Smoking in Pregnancy: Genome-wide Consortium Meta-analysis. Am J Hum Genet.

[4] Shah S, McRae AF, Marioni RE, Harris SE, Gibson J, Henders AK (2014). Genetic and environmental exposures constrain epigenetic drift over the human life course. Genome Res.

[5] Horvath S (2013). DNA methylation age of human tissues and cell types. Genome Biol.

[6] Heyn H, Li N, Ferreira HJ, Moran S, Pisano DG, Gomez A (2012). Distinct DNA methylomes of newborns and centenarians. Proc Natl Acad Sci.

[7] Alisch RS, Barwick BG, Chopra P, Myrick LK, Satten GA, Conneely KN. Age-associated DNA methylation in pediatric populations. Genome Res [Internet]. 2012. 22. Available from: http://dx.doi.org/10.1101/gr.125187.111. 10.1101/gr.125187.111 PMC3317145 22300631

[8] Acevedo N, Reinius LE, Vitezic M, Fortino V, Söderhäll C, Honkanen H (2015). Age-associated DNA methylation changes in immune genes, histone modifiers and chromatin remodeling factors within 5 years after birth in human blood leukocytes. Clin Epigenetics.

[9] Reynolds LM, Taylor JR, Ding J, Lohman K, Johnson C, Siscovick D, et al. Age-related variations in the methylome associated with gene expression in human monocytes and T cells. Nat Commun [Internet]. 2014. 5. Available from: http://dx.doi.org/10.1038/ncomms6366. 10.1038/ncomms6366 PMC4280798 25404168

[10] Bousquet J, Anto JM, Akdis M, Auffray C, Keil T, Momas I, et al. Paving the way of systems biology and precision medicine in allergic diseases: The MeDALL success story. Allergy [Internet]. 2016. Available from: http://dx.doi.org/10.1111/all.12880. 10.1111/all.12880 PMC5248602 26970340

[11] Houseman E, Accomando W, Koestler D, Christensen B, Marsit C, Nelson H (2012). DNA methylation arrays as surrogate measures of cell mixture distribution. BMC Bioinformatics.

[12] Dev Borman A, Ocaka LA, Mackay DS, Ripamonti C, Henderson RH, Moradi P (2012). Early Onset Retinal Dystrophy Due to Mutations in LRAT: Molecular Analysis and Detailed Phenotypic Study Early Onset Retinal Dystrophy Due to Mutations in LRAT. Invest Ophthalmol Vis Sci.

[13] Berkel S, Marshall CR, Weiss B, Howe J, Roeth R, Moog U (2010). Mutations in the SHANK2 synaptic scaffolding gene in autism spectrum disorder and mental retardation. Nat Genet.

[14] Kundaje A, Meuleman W, Ernst J, Bilenky M, Yen A, Roadmap Epigenomics Consortium (2015). Integrative analysis of 111 reference human epigenomes. Nature.

[15] Rakyan VK, Down TA, Maslau S, Andrew T, Yang TP, Beyan H (2010). Human aging-associated DNA hypermethylation occurs preferentially at bivalent chromatin domains. Genome Res.

[16] Voigt P, Tee W-W, Reinberg D (2013). A double take on bivalent promoters. Genes Dev.

[17] Creyghton MP, Cheng AW, Welstead GG, Kooistra T, Carey BW, Steine EJ (2010). Histone H3K27ac separates active from poised enhancers and predicts developmental state. Proc Natl Acad Sci.

[18] Fehrmann RSN, Karjalainen JM, Krajewska M, Westra H-J, Maloney D, Simeonov A (2015). Gene expression analysis identifies global gene dosage sensitivity in cancer. Nat Genet.

[19] Gokhman D, Lavi E, Prufer K, Fraga MF, Riancho JA, Kelso J (2014). Reconstructing the DNA Methylation Maps of the Neandertal and the Denisovan. Science.

[20] Chen L, Page GP, Mehta T, Feng R, Cui X (2009). Single Nucleotide Polymorphisms Affect both Cis- and Trans-eQTLs. Genomics.

[21] Gaunt TR, Shihab HA, Hemani G, Min JL, Woodward G, Lyttleton O (2016). Systematic identification of genetic influences on methylation across the human life course. Genome Biol.

[22] Punzo D, Errico F, Cristino L, Sacchi S, Keller S, Belardo C (2016). Age-Related Changes in d-Aspartate Oxidase Promoter Methylation Control Extracellular d-Aspartate Levels and Prevent Precocious Cell Death during Brain Aging. J Neurosci.

[23] Solomon BD, Nguyen A-D, Bear KA, Wolfsberg TG (2013). Clinical Genomic Database. Proc Natl Acad Sci.

[24] Koppelman GH, Nawijn MC (2011). Recent advances in the epigenetics and genomics of asthma: Curr. Opin Allergy Clin Immunol.

[25] Pinart M, Benet M, Annesi-Maesano I, von Berg A, Berdel D, Carlsen KCL (2014). Comorbidity of eczema, rhinitis, and asthma in IgE-sensitised and non-IgE-sensitised children in MeDALL: a population-based cohort study. Lancet Respir Med.

[26] Huh I, Zeng J, Park T, Yi S (2013). DNA methylation and transcriptional noise. Epigenetics Chromatin.

[27] Westra H-J, Peters MJ, Esko T, Yaghootkar H, Schurmann C, Kettunen J (2013). Systematic identification of trans eQTLs as putative drivers of known disease associations. Nat Genet.

[28] Errico F, Nisticò R, Napolitano F, Oliva AB, Romano R, Barbieri F (2011). Persistent increase of d-aspartate in d-aspartate oxidase mutant mice induces a precocious hippocampal age-dependent synaptic plasticity and spatial memory decay. Neurobiol Aging.

[29] Errico F, Di Maio A, Marsili V, Squillace M, Vitucci D, Napolitano F (2013). Bimodal effect of D-aspartate on brain aging processes: insights from animal models. J Biol Regul Homeost Agents.

[30] Liu Y, Aryee MJ, Padyukov L, Fallin MD, Hesselberg E, Runarsson A (2013). Epigenome-wide association data implicate DNA methylation as an intermediary of genetic risk in rheumatoid arthritis. Nat Biotech.

[31] Hanson M, Gluckman P (2011). Developmental origins of noncommunicable disease: population and public health implications. Am J Clin Nutr.

[32] Wijga AH, Kerkhof M, Gehring U, de Jongste JC, Postma DS, Aalberse RC (2014). Cohort profile: The Prevention and Incidence of Asthma and Mite Allergy (PIAMA) birth cohort. Int J Epidemiol.

[33] Peters MJ, Joehanes R, Pilling LC, Schurmann C, Conneely KN, Powell J (2015). The transcriptional landscape of age in human peripheral blood. Nat Commun.

[34] Kull I, Melen E, Alm J, Hallberg J, Svartengren M, van Hage M (2010). Breast-feeding in relation to asthma, lung function, and sensitization in young schoolchildren. J Allergy Clin Immunol.

[35] Baïz N, Slama R, Béné M-C, Charles M-A, Kolopp-Sarda M-N, Magnan A (2011). Maternal exposure to air pollution before and during pregnancy related to changes in newborn’s cord blood lymphocyte subpopulations. The EDEN study cohort. BMC Pregnancy Childbirth.

[36] Guxens M, Ballester F, Espada M, Fernández MF, Grimalt JO, Ibarluzea J (2012). Cohort Profile: The INMA-INfancia y Medio Ambiente-(Environment and Childhood) Project. Int J Epidemiol.

[37] Aryee MJ, Jaffe AE, Corrada-Bravo H, Ladd-Acosta C, Feinberg AP, Hansen KD (2014). Minfi: a flexible and comprehensive Bioconductor package for the analysis of Infinium DNA methylation microarrays. Bioinformatics.

[38] Chen Y, Lemire M, Choufani S, Butcher DT, Grafodatskaya D, Zanke BW (2013). Discovery of cross-reactive probes and polymorphic CpGs in the Illumina Infinium HumanMethylation450 microarray. Epigenetics.

[39] Pidsley R, Wong CC Y, Volta M, Lunnon K, Mill J, Schalkwyk LC. A data-driven approach to preprocessing Illumina 450 K methylation array data. BMC Genomics [Internet]. 2013. 14. Available from: http://dx.doi.org/10.1186/1471-2164-14-293. 10.1186/1471-2164-14-293 PMC3769145 23631413

[40] Gagnon-Bartsch JA, Speed TP (2012). Using control genes to correct for unwanted variation in microarray data. Biostat Oxf Engl.

[41] Zhang B, Gaiteri C, Bodea L-G, Wang Z, McElwee J, Podtelezhnikov AA (2013). Integrated Systems Approach Identifies Genetic Nodes and Networks in Late-Onset Alzheimer’s Disease. Cell.

[42] the EArly Genetics and Lifecourse Epidemiology (EAGLE) Eczema Consortium (2015). Multi-ancestry genome-wide association study of 21,000 cases and 95,000 controls identifies new risk loci for atopic dermatitis. Nat Genet.

[43] Deelen P, Bonder M, van der Velde K, Westra H-J, Winder E, Hendriksen D (2014). Genotype harmonizer: automatic strand alignment and format conversion for genotype data integration. BMC Res Notes.

[44] Shabalin AA. Matrix eQTL: ultra fast eQTL analysis via large matrix operations. Bioinformatics. [Internet]. 2012. 28. Available from: http://dx.doi.org/10.1093/bioinformatics/bts163. 10.1093/bioinformatics/bts163 PMC3348564 22492648

[45] Willer CJ, Li Y, Abecasis GR (2010). METAL: fast and efficient meta-analysis of genomewide association scans. Bioinformatics.

[46] An integrated map of genetic variation from 1,092 human genomes. Nature. 2012;491:56–65. 10.1038/nature11632 PMC3498066 23128226

[47] McLean CY, Bristor D, Hiller M, Clarke SL, Schaar BT, Lowe CB (2010). GREAT improves functional interpretation of cis-regulatory regions. Nat Biotechnol.

[48] Pruim RJ, Welch RP, Sanna S, Teslovich TM, Chines PS, Gliedt TP (2010). LocusZoom: regional visualization of genome-wide association scan results. Bioinformatics.

