# Supplementary material for: The emerging landscape of dynamic DNA methylation in early childhood
Source: BMC Genomics. 2017 Jan 5;18:25. doi: 10.1186/s12864-016-3452-1 (PMC5217260; doi:10.1186/s12864-016-3452-1)
Supplement: Additional file 1: Tables S1-S10. — (DOCX 1279 kb) [file 12864_2016_3452_MOESM1_ESM.docx]

**The emerging landscape of dynamic DNA methylation in early childhood**

Cheng-Jian Xu, Marc Jan Bonder, Cilla Söderhäll, Mariona Bustamante, Nour Baïz, Ulrike Gehring, Soesma A. Jankipersadsing, Peter van der Vlies, Cleo van Diemen, Bianca Rijkom, Jocelyne Just, Inger Kull, Juha Kere, Josep Maria Antó, Jean Bousquet, Alexandra Zhernakova, Cisca Wijmenga, Isabella Annesi-Maesano, Jordi Sunyer, Erik Melén, Yang Li, Dirkje S. Postma & Gerard H. Koppelman

**Supplementary tables**

Table S1. Numbers and basic characteristics of participants included in this study.

Table S2. Number of a-DMSs between Ages 0-4/5 and Ages 4-8.

Table S3. Comparsion of a-DMSs with two published pediatric studies.

Table S4. a-DMSs annotated to the 5’ *HOXD* cluster genes.

Table S5. a-DMSs annotated to the *MEIS1* gene.

Table S6. a-DMSs significantly associated to maternal smoking.

Table S7. The number of a-DMSs associated with maternal smoking (*P*<0.05).

Table S8. a-DMS-associated genes are enriched for disease genes.

Table S9. a-DMS sites associated with asthma (FDR *P*<0.05).

Table S10. Numbers and basic characteristics of participants included in the asthma study.

Table S1. Numbers and basic characteristics of participants included in this study.

|  | EDEN | INMA | BAMSE | PIAMA |
| --- | --- | --- | --- | --- |
| Data available: N | 136 | 133 | 179 | 184 |
| Age 4/5: mean (SD) | 5.65 (0.10) | 4.34 (0.21) | 4.25 (0.19) | 4.08 (0.21) |
| Age 8: mean (SD) | - | - | 8.33 (0.41) | 8.07 (0.27) |
| Male (%) | 56.6 | 48.1 | 50.2 | 49.5 |
| Maternal smoking (%) | 13.2 | 8.33 | 6.14 | 8.15 |
| Asthma at 4 years (%) | 17.6 | 0.0 | 19.2 | 13.6 |
| Asthma at 8 years (%) | - | - | 13.6 | 8.7 |
| Genome-wide SNP data: N | - | 114 | 49 | 181 |

SNP: Single Nucleotide Polymorphisms

Table S2. Number of a-DMSs (age-differential methylation CpG sites) between Ages 0-4/5 and Ages 4-8.

| Direction of effect  Ages 0-4/5 | Direction of effect  Ages 4-8 | Number of  CpG sites |
| --- | --- | --- |
| - | - | 9,704 |
| + | + | 4,446 |
| - | + | 355 |
| + | - | 1,024 |

Table S3. Comparsion of a-DMSs in this study with two published pediatric studies.

|  | Alisch et al^1^. | Acevedo et al^2^. |
| --- | --- | --- |
| Array | Illumina 27k | Illumina 450k |
| Individuals | 396 boys | 10 girls |
| Type | Cross-sectional | Longitudinal |
| Thresholds | FDR<0.01 | Bonferroni<0.01 |
| Age | 3-17 years | 3,6,23,34,36,48,60 months |
| Overlapping da-DMSs | 392/675 (58%)* | 299/464 (64%)** |
| Overlapping ia-DMSs | 94/185 (51%)* | 177/330 (54%)** |

* There are 675 da-DMSs and 185 ia-DMSs present in the Illumina 27K platform.

** In Acevedo’s study, 464 da-DMSs and 330 ia-DMSs were reported.

1. Alisch, R. S. *et al.* Age-associated DNA methylation in pediatric populations. *Genome Res* **22,** (2012).

2. Acevedo, N. *et al.* Age-associated DNA methylation changes in immune genes, histone modifiers and chromatin remodeling factors within 5 years after birth in human blood leukocytes. *Clin. Epigenetics* **7,** 1–20 (2015).

Table S4. Identified a-DMSs annotated to the 5’ *HOXD* cluster genes.

| ILMNID | CHR | MAPINFO | Gene | Group | CpG_island |
| --- | --- | --- | --- | --- | --- |
| cg04730882 | 2 | 176986535 | *HOXD9* | TSS1500 | Island |
| cg14410016 | 2 | 176986659 | *HOXD9* | TSS1500 | Island |
| cg18115040 | 2 | 176981328 | *HOXD10* | TSS200 | Island |
| cg05979020 | 2 | 176981336 | *HOXD10* | TSS200 | Island |
| cg05500840 | 2 | 176972068 | *HOXD11* | TSS200 | Island |
| cg24633978 | 2 | 176972581 | *HOXD11* | 1stExon | N_Shore |
| cg03964958 | 2 | 176964720 | *HOXD12* | 1stExon | Island |
| cg04415176 | 2 | 176957842 | *HOXD13* | 1stExon | Island |

ILMNID: Unique CpG locus identifier from the Illumina CG database

CHR: Chromosome - genome build 37

MAPINFO: Coordinates - genome build 37

Gene: Gene name (UCSC)

Group: Gene region feature category (UCSC)

CpG_island: Relationship to Canonical CpG Island

Table S5. Identified a-DMSs annotated to the *MEIS1* gene.

| ILMNID | CHR | MAPINFO | Gene | Group | CpG_Island |
| --- | --- | --- | --- | --- | --- |
| cg12055515 | 2 | 66735203 | *MEIS1* | Body | Open sea |

Table S6. Nine a-DMSs significantly associated to maternal smoking.

| ILMNID | CHR | Direction | Gene | Beta | P-value | Age |
| --- | --- | --- | --- | --- | --- | --- |
| cg09836827 | 12 | da-DMS | *VWF* | -0.015 | 4.59 × 10^- 8^ | 0-4/5 |
| cg10574566 | 1 | da-DMS |  | -0.027 | 6.46 × 10^- 6^ | 0-4/5 |
| cg25707298 | 10 | da-DMS |  | -0.017 | 6.48 × 10^-6^ | 0-4/5 |
| cg23418219 | 7 | da-DMS | *CREB5* | -0.028 | 9.58 × 10^- 6^ | 0-4/5 |
| cg09706133 | 15 | da-DMS | *ITGA11* | -0.021 | 1.78 × 10^-5^ | 0-4/5 |
| cg00849025 | 11 | da-DMS | *OR5B3* | -0.018 | 2.35 × 10^- 5^ | 0-4/5 |
| cg25065097 | 13 | da-DMS | *RCBTB1* | -0.024 | 2.45 × 10^- 5^ | 0-4/5 |
| cg26191447 | 2 | da-DMS |  | -0.036 | 2.63 × 10^- 5^ | 0-4/5 |
| cg04625975 | 10 | da-DMS | *HNRNPA3P1* | -0.013 | 1.52 × 10^-6^ | 4-8 |

da-DMS: decreasing with age-differential methylation sites

Table S7. The number of a-DMSs associated with maternal smoking (*P*<0.05).

|  |  | Ages 0-4/5 |  | Ages 4-8 |
| --- | --- | --- | --- | --- |
| a-DMSs  da-DMSs |  | 971  787 |  | 619  422 |
| Negatively associated a-DMSs  Negatively associated da-DMSs |  | 785  721 |  | 295  203 |
|  |  |  |  |  |

Table S8. a-DMS-associated genes are enriched for disease genes, annotated by the Clinical Genomic Database (accessed 27-Feb-2016).

|  |  | 450k Genome-wide |  | a-DMSs |
| --- | --- | --- | --- | --- |
| Total number of genes |  | 19,942 |  | 5,900 |
| Number of disease-linked genes |  | 2,820 |  | 997 |
| Disease CGD%  Enrichment P-value |  | 14.1%  4.9 × 10^-6^ |  | 16.9% |
|  |  |  |  |  |

Enrichment P-value by Fisher’s exact test.

Table S9. a-DMSs associated with asthma (FDR *P*<0.05)

| ILMNID | CHR | MAPINFO | GENE | Group | P-value | Age |
| --- | --- | --- | --- | --- | --- | --- |
| cg22971191 | 13 | 103719706 | *SLC10A2* | TSS1500 | 4.1 × 10^-7^ | 4 |
| cg05712073 | 12 | 6795171 | *ZNF384;* | 5'UTR; | 9.3 × 10^-7^ | 4 |
| cg18515031 | 10 | 73988469 | *C10orf104* | Body | 1.3 × 10^-6^ | 4 |
| cg02977254 | 11 | 122774810 | *C11orf63;* | Body | 1.8 × 10^-6^ | 8 |

Table S10. Numbers and basic characteristics of participants included in the asthma study.

|  | |  | EDEN | |  | INMA | | | BAMSE | PIAMA |
| --- | --- | --- | --- | --- | --- | --- | --- | --- | --- | --- |
| Data available at age 4/5 | |  | 147 | |  | 211 | | | 244 | 214 |
| Data available at age 8  Age 4/5 mean (SD) | |  | *NA*  5.64(0.12) | |  | *NA*  4.36(0.21) | | | 232  4.26(0.19) | 191  4.08(0.20) |
| Age 8 mean (SD) | |  | *NA* | |  | *NA* | | | 8.32(0.40) | 8.07(0.27) |
| Male sex at age 4/5 (%) | |  | 59.2% | |  | 52.1% | | | 54.1% | 54.2% |
| Male sex at age 8 (%) | |  | *NA* | |  | *NA* | | | 53.0% | 50.3% |
| Asthma at 4/5 years (%) | |  | 23.1% | |  | 16.6% | | | 40.0% | 18.7% |
| Asthma at 8 years (%) | |  | *NA* | |  | *NA* | | | 28.4% | 12.0% |
|  |  | | |  | | |  |  | | |

*NA: not applicable*
