# Supplementary material for: The emerging landscape of dynamic DNA methylation in early childhood
Source: BMC Genomics. 2017 Jan 5;18:25. doi: 10.1186/s12864-016-3452-1 (PMC5217260; doi:10.1186/s12864-016-3452-1)
Supplement: Additional file 2: Figures S1-S7. — (DOCX 45 kb) [file 12864_2016_3452_MOESM2_ESM.docx]

**The emerging landscape of dynamic DNA methylation in early childhood**

Cheng-Jian Xu, Marc Jan Bonder, Cilla Söderhäll, Mariona Bustamante, Nour Baïz, Ulrike Gehring, Soesma A. Jankipersadsing, Peter van der Vlies, Cleo van Diemen, Bianca Rijkom, Jocelyne Just, Inger Kull, Juha Kere, Josep Maria Antó, Jean Bousquet, Alexandra Zhernakova , Cisca Wijmenga, Isabella Annesi-Maesano, Jordi Sunyer, Erik Melén, Yang Li, Dirkje S. Postma & Gerard H. Koppelman

**Supplementary figures**

Figure S1. Overall correlations of methylation levels in each cohort between age 0-4/5 and age 4-8.

Figure S2. Volcano plot representing the methylation difference between ages 0-4/5 and ages 4-8 after adjusting for sex, cell counts and batch variables.

Figure S3. Venn diagram showing the number of a-DMSs between ages 0-4/5 and ages 4-8.

Figure S4. The effect of maternal smoking on methylation change of cg09836827(*VWF*) in the age 0-4/5 group.

Figure S5. Proposed mechanism of dMeQTLs of rs93200331-cg00804078 in the *DDO* gene.

Figure S6. Exposure to maternal smoking effect on age estimation based on methylation by using Horvath’s “epigenetic clock” a) age 4/5 samples b) age 8 samples.

Figure S7. Predicted age vs. reported age using Horvath’s “epigenetic clock”. a) age 4. b) age 5. c) age 8.

Figure S1. Overall correlations (Spearman rho correlation) of methylation levels in each cohort between age 0-4/5 (EDEN, INMA) and age 4-8 (BAMSE, PIAMA).


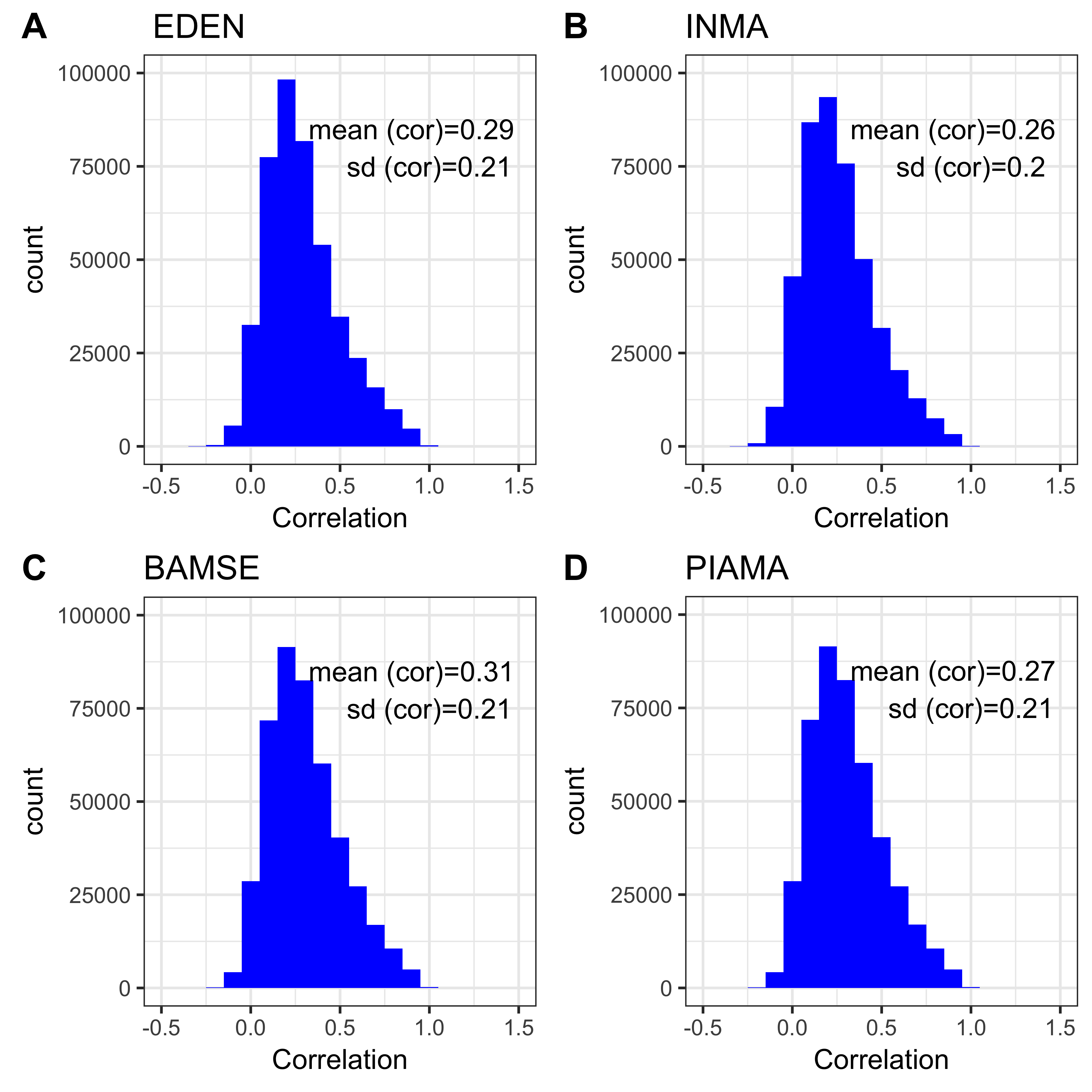


Figure S2. Volcano plot of −log10 (p-value) against regression coefficient, representing the methylation difference between a) ages 0-4/5 and b) ages 4-8, after adjusting for sex, cell counts and batch variables. The dashed red lines represent the threshold used for statistical cutoff (Bonferroni-adjusted *P*=0.05).


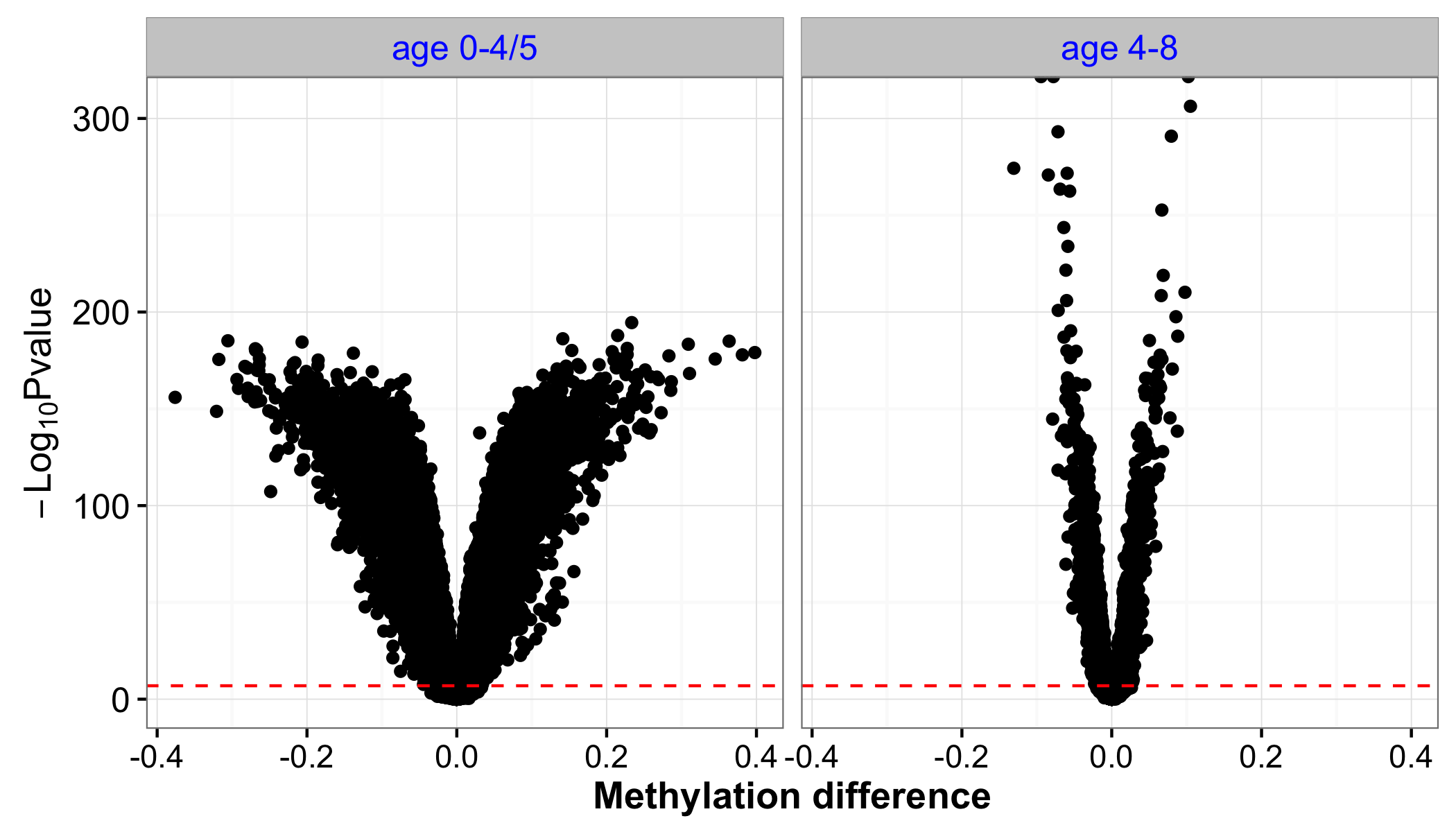


Figure S3. Venn diagram showing the number of a-DMSs in ages 0-4/5 and ages 4-8. There are 15,529 CpG site a-DMSs that overlap between the two groups.


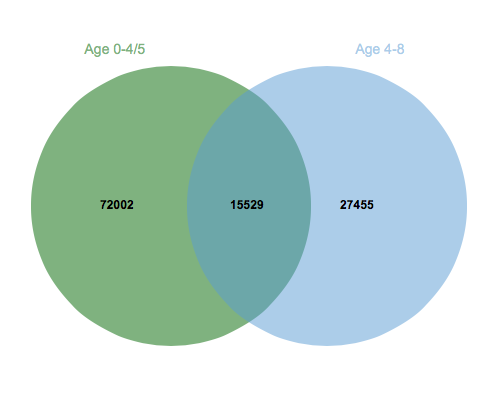


Figure S4. The effect of maternal smoking on methylation change of cg09836827(*VWF*) in the age 0-4/5 group.


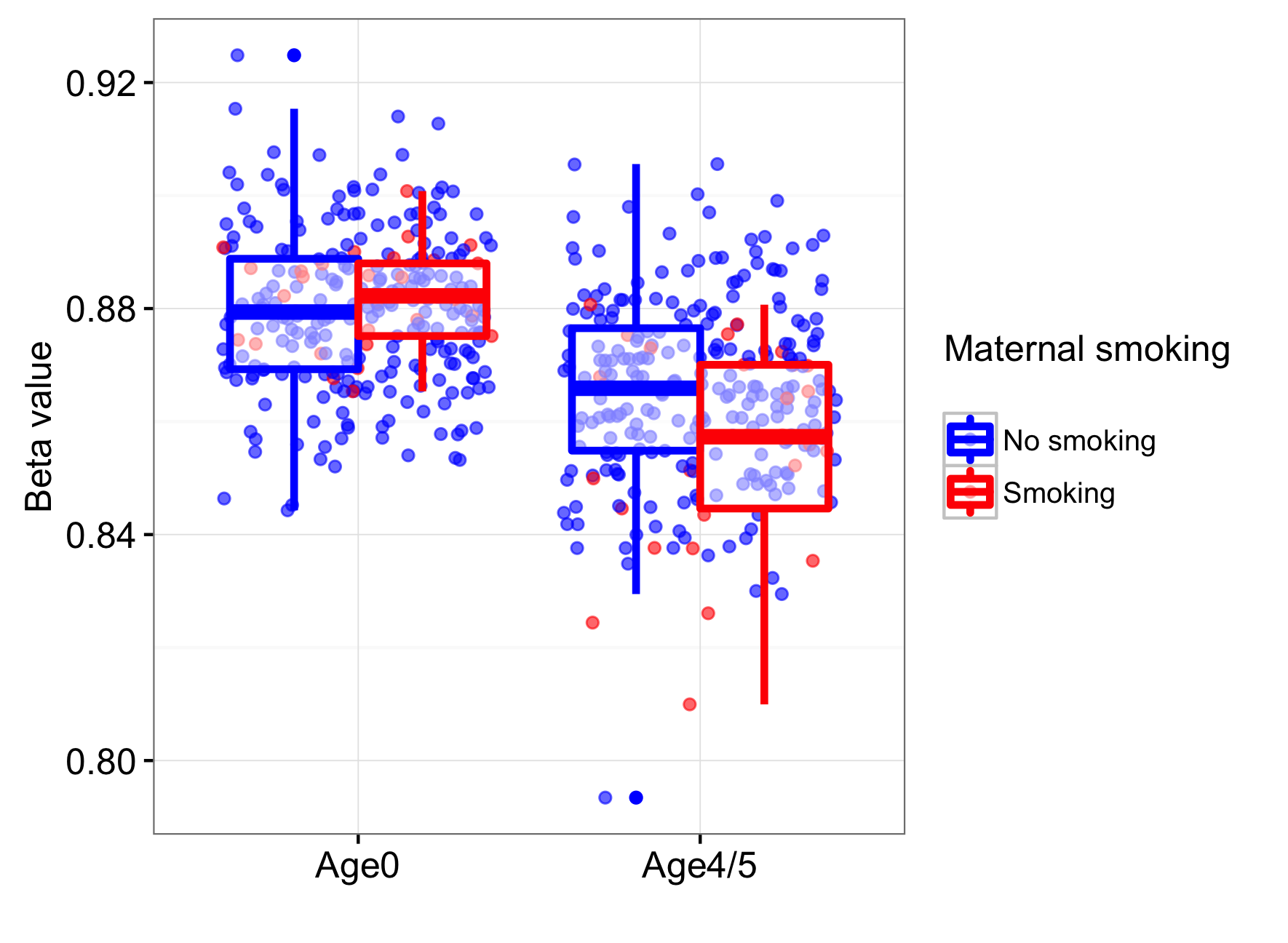


Figure S5. Proposed mechanism of dMeQTLs of rs93200331-cg00804078 in the *DDO* gene.


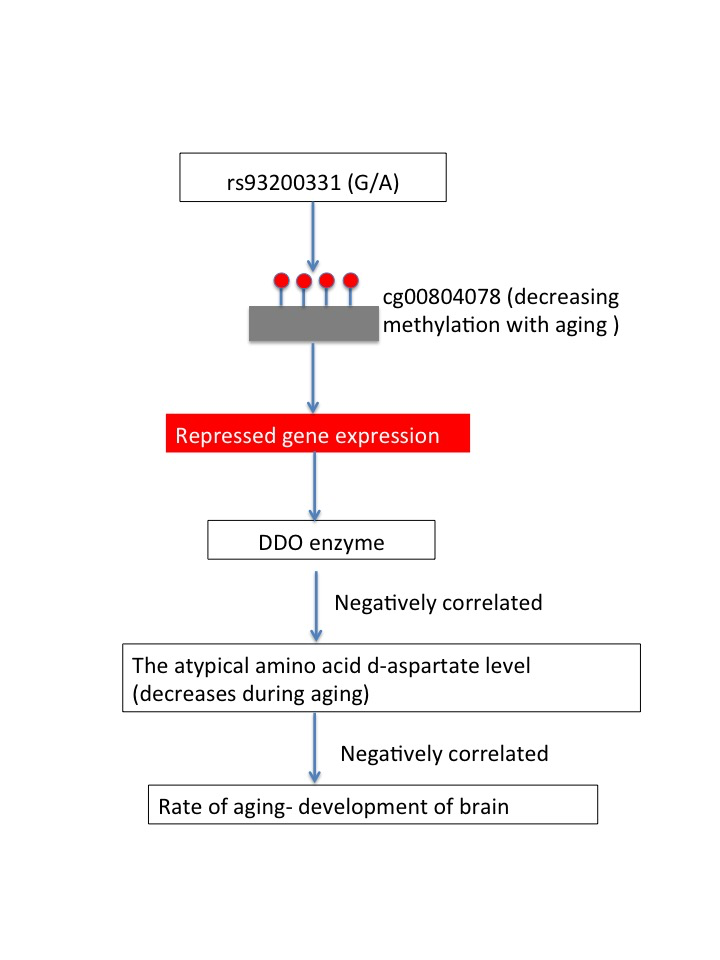


Figure S6. Exposure to maternal smoking effect on age estimation based on methylation by using Horvath’s “epigenetic clock” a) age 4/5 samples b) age 8 samples.


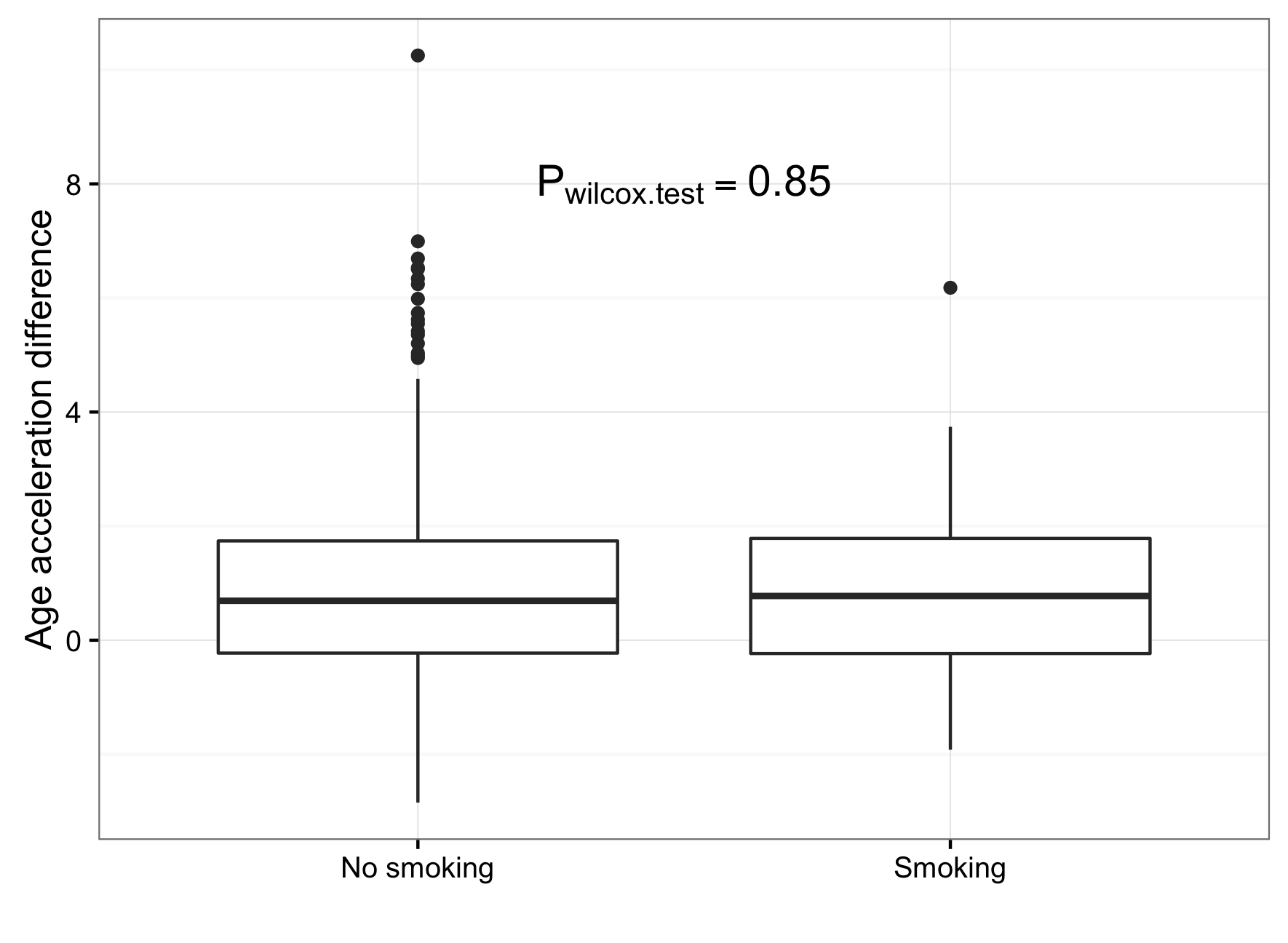


a)

b)


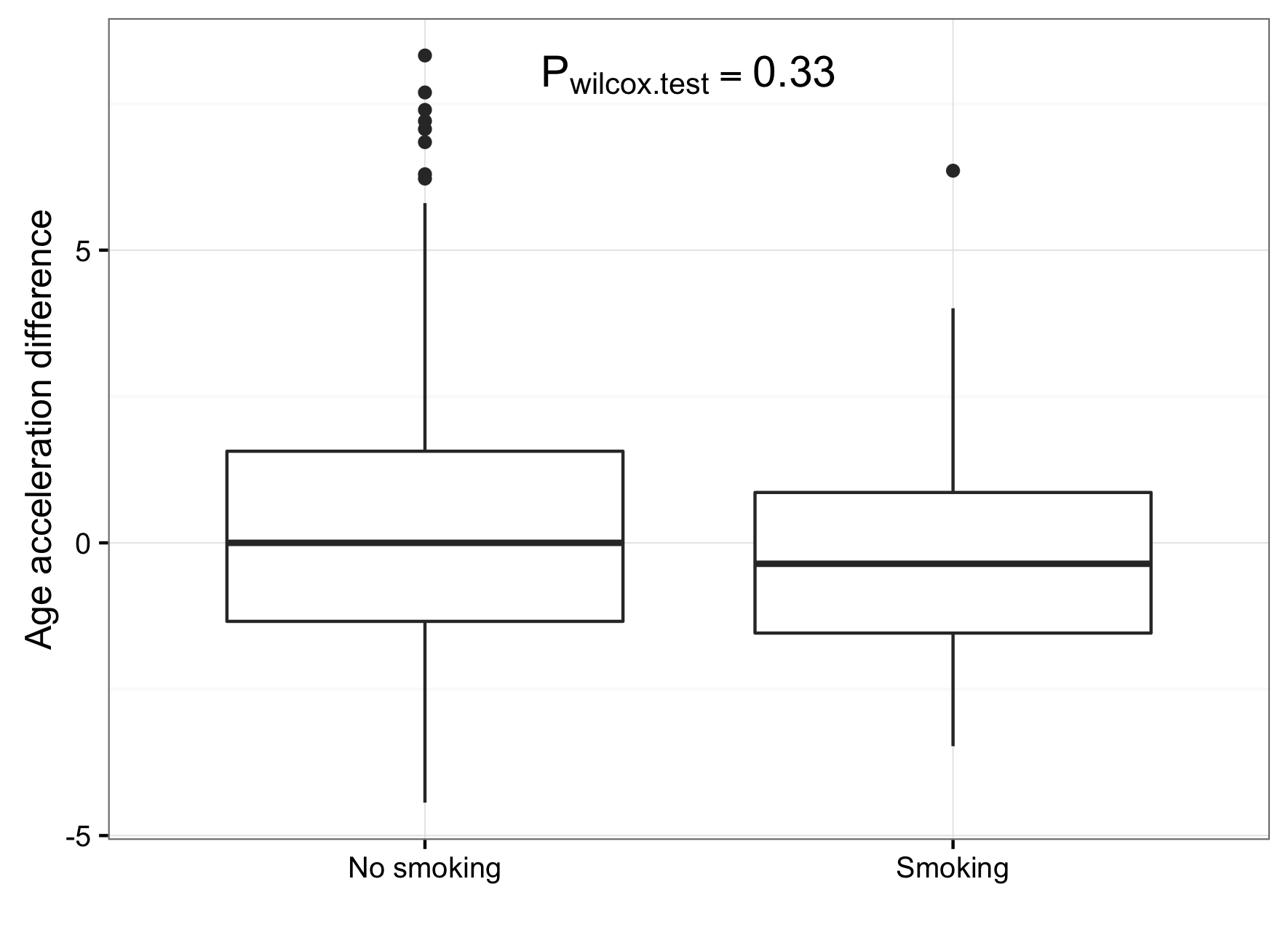


Figure S7. Predicted age vs. reported age using Horvath’s “epigenetic clock”. a) age 4. b) age 5. c) age 8.


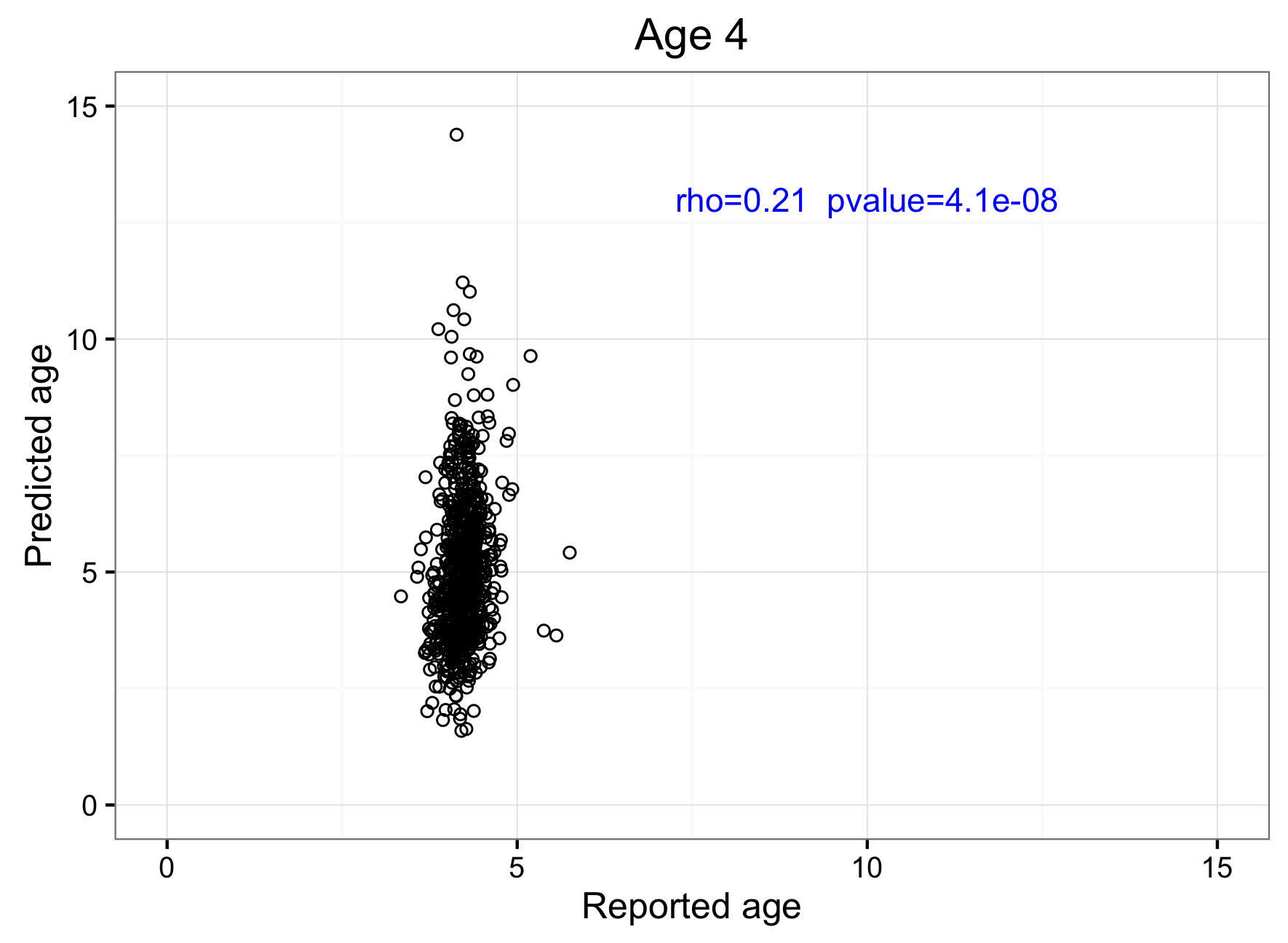


a)


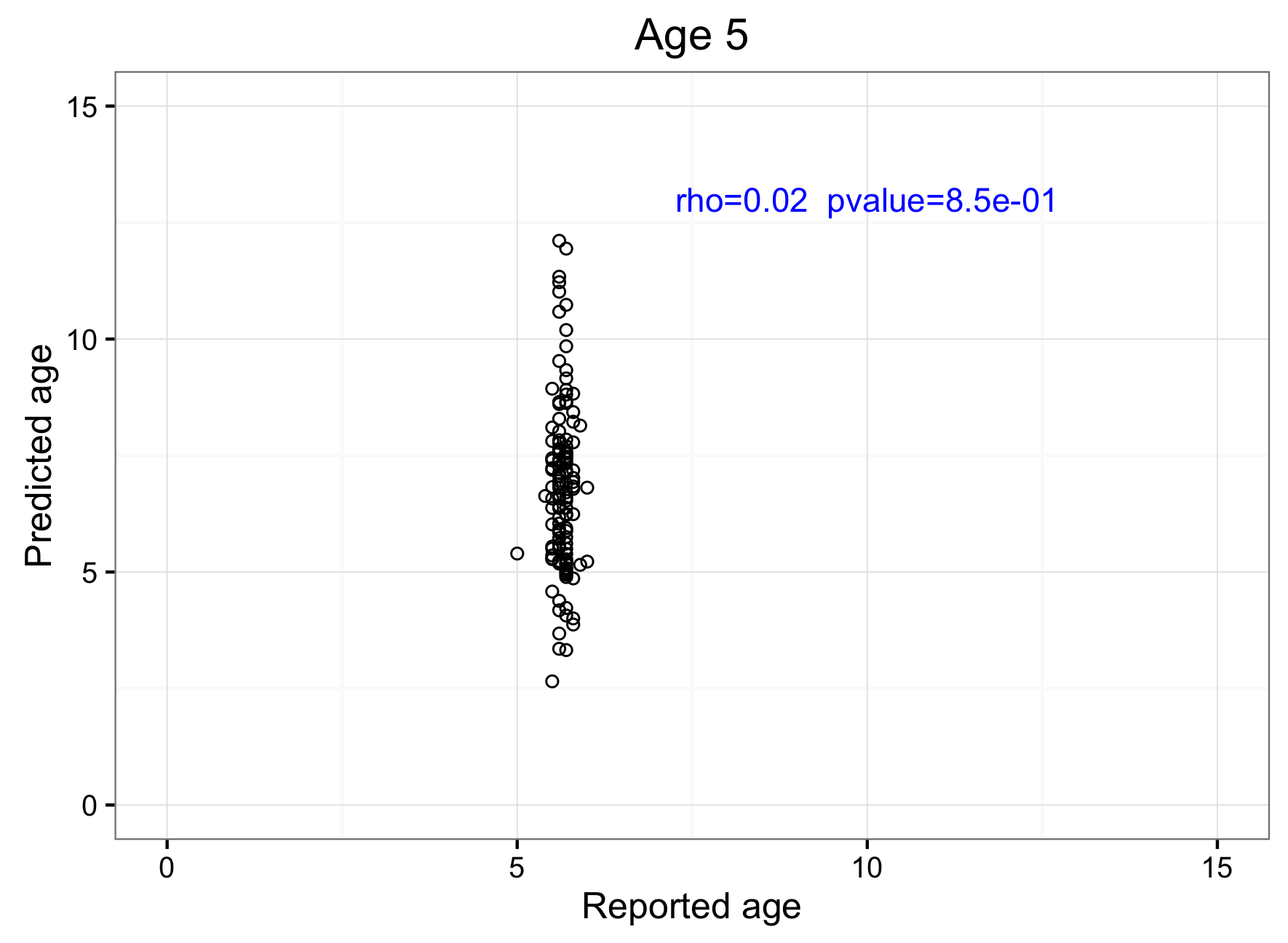


b)


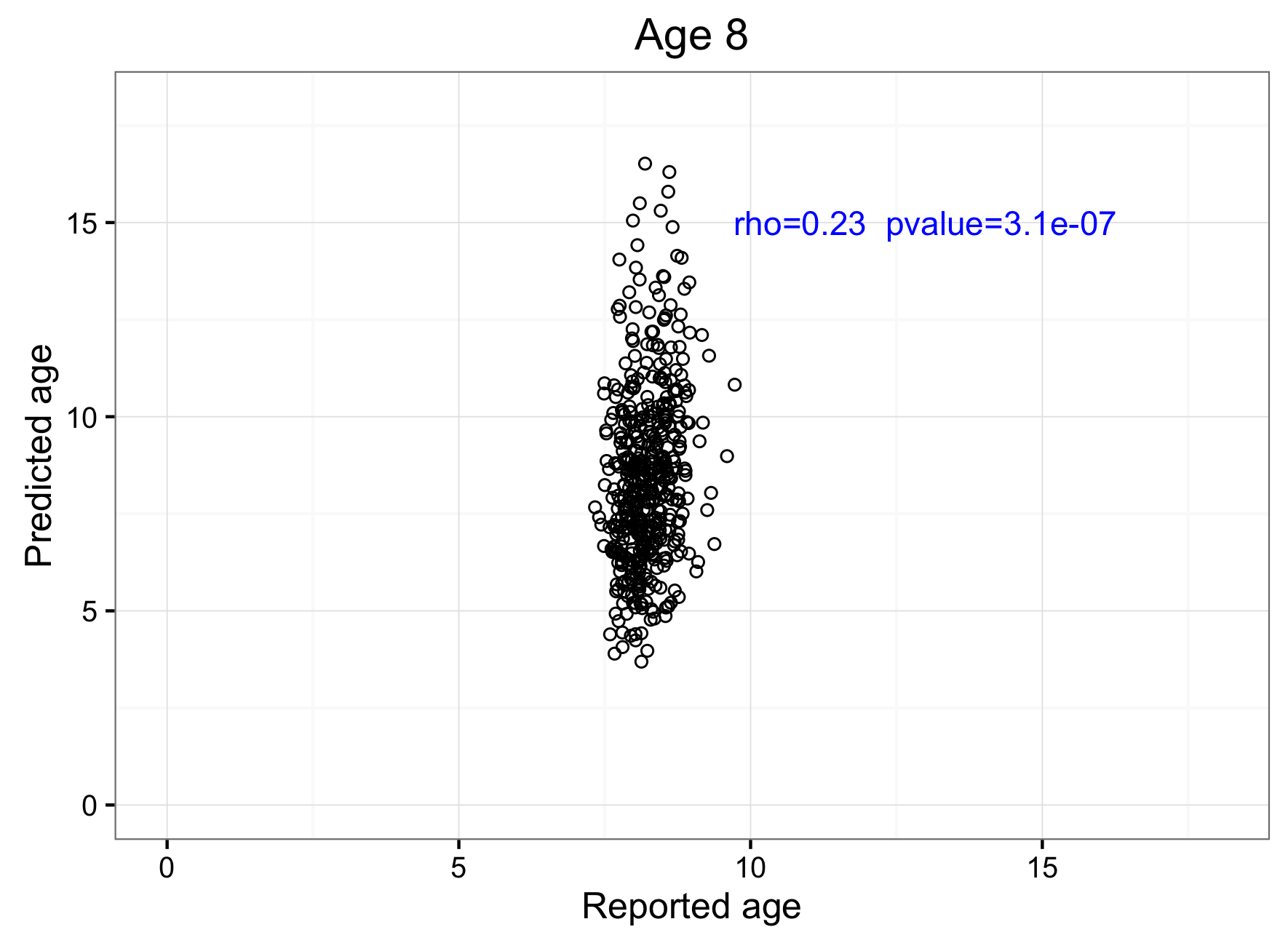


c)
